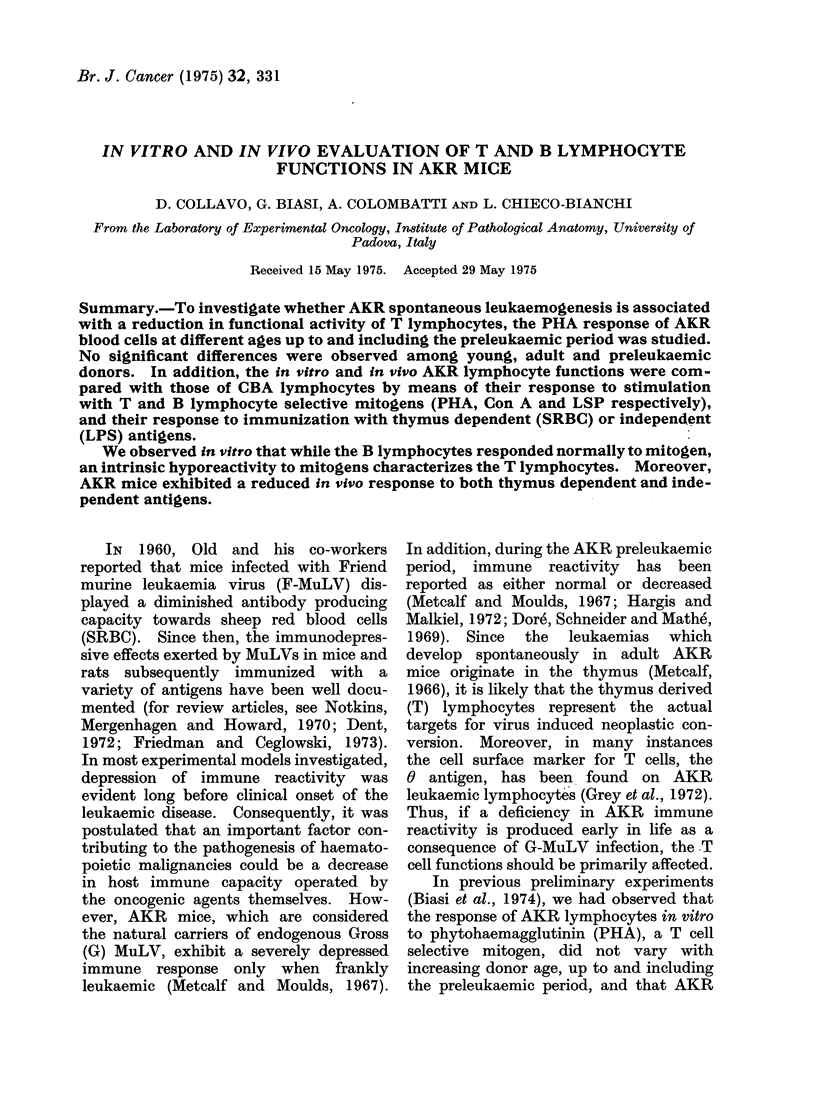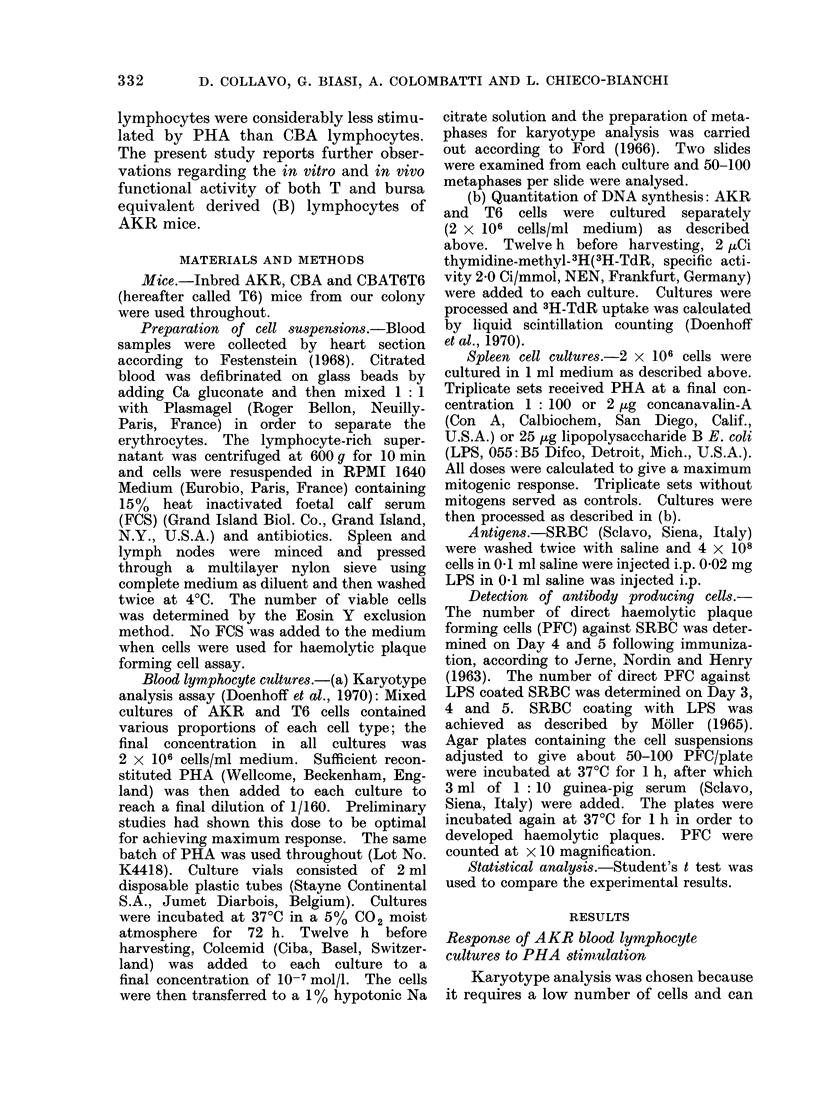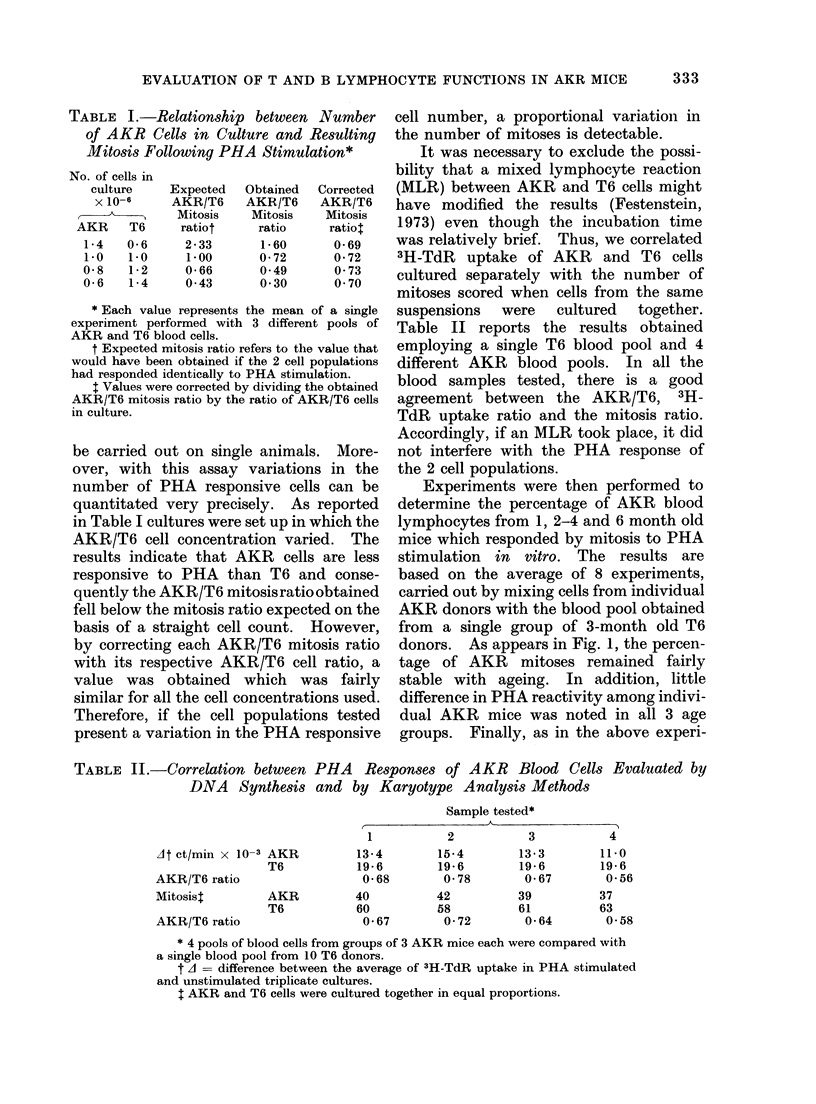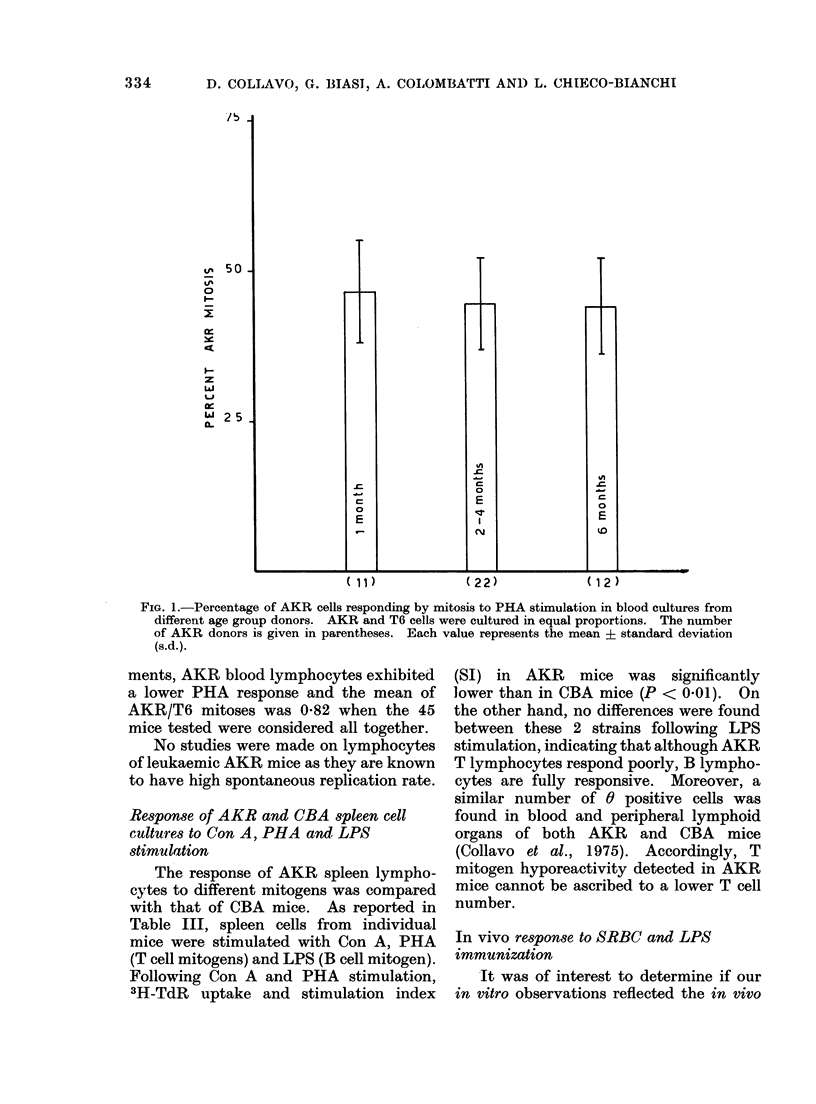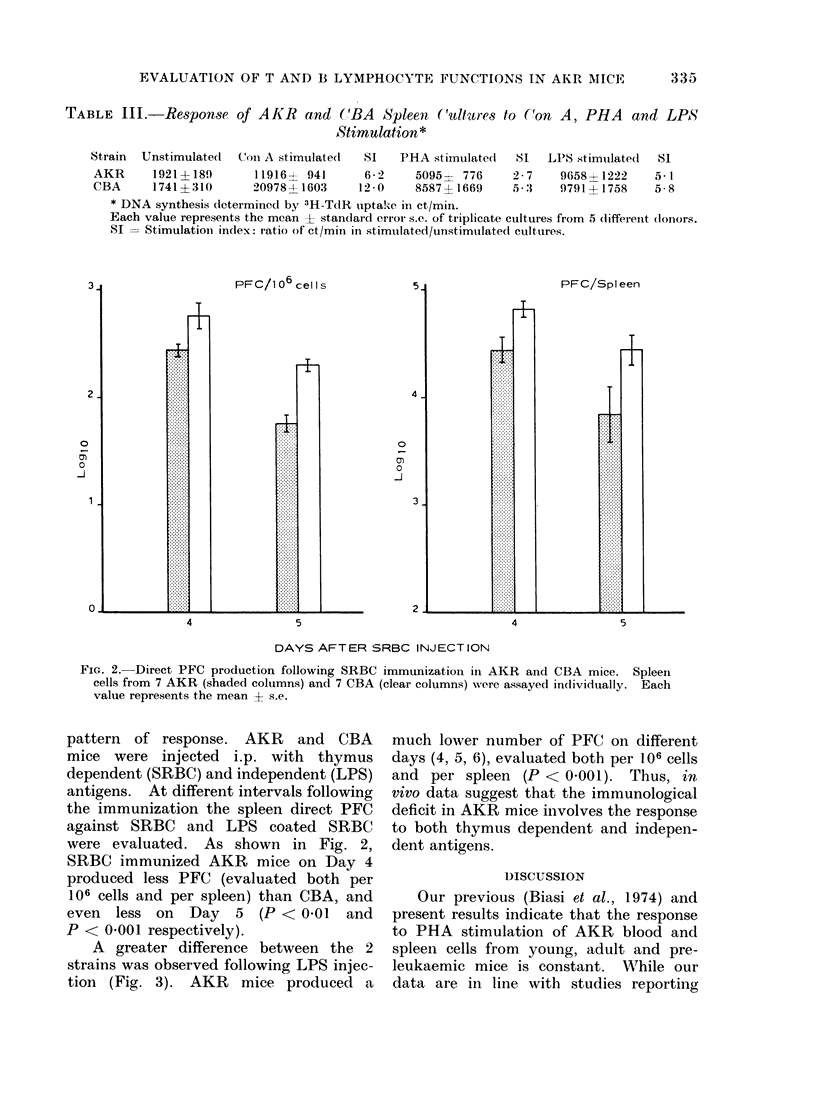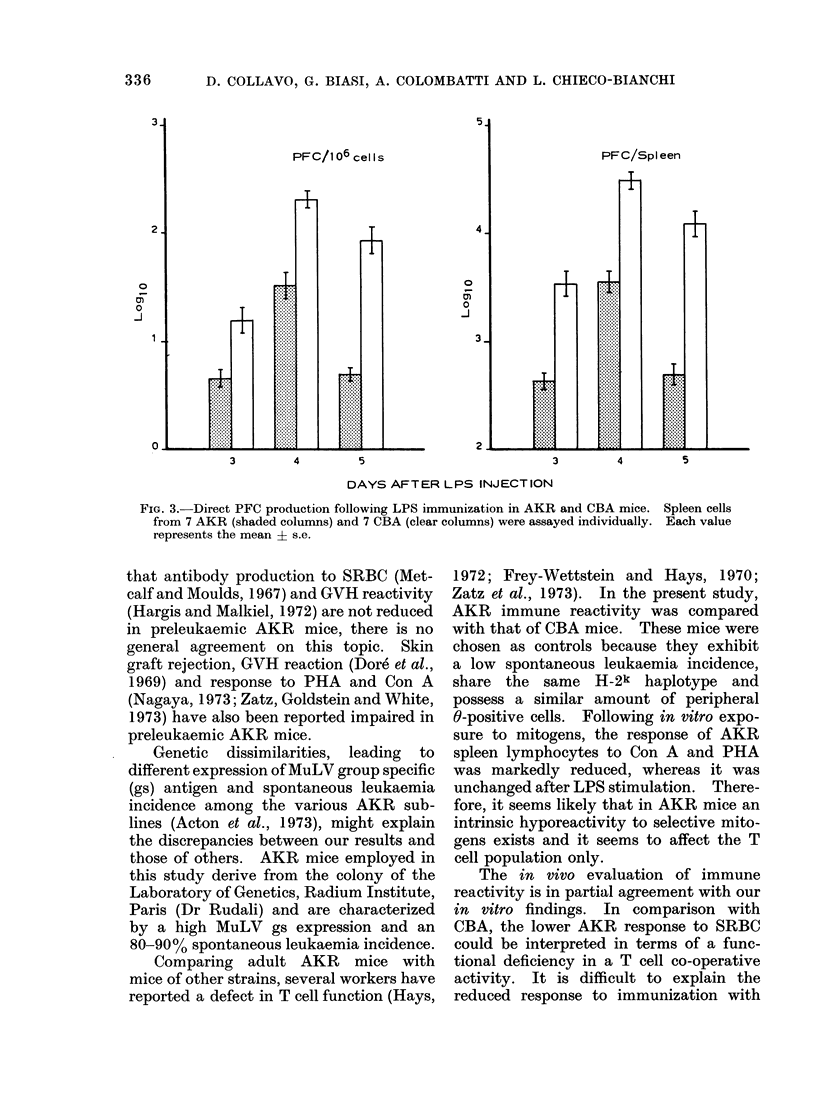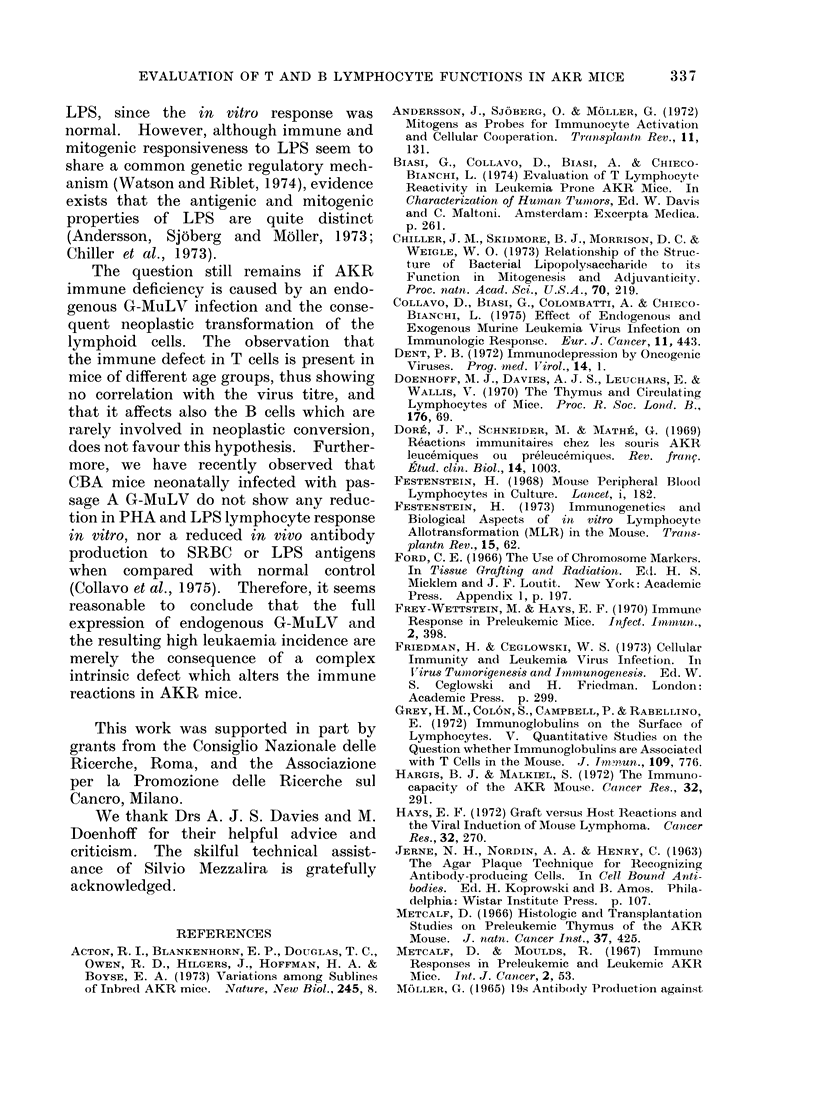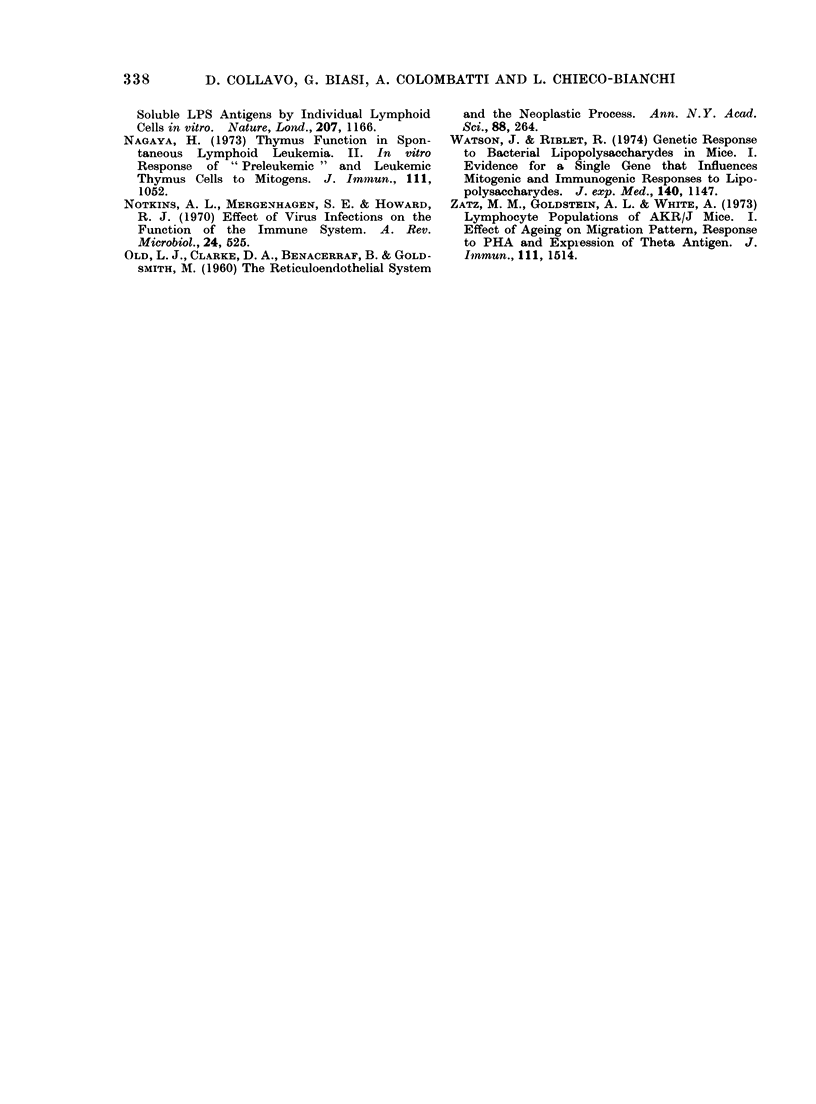# In vitro and in vivo evaluation of T and B lymphocyte functions in AKR mice.

**DOI:** 10.1038/bjc.1975.231

**Published:** 1975-09

**Authors:** D. Collavo, G. Biasi, A. Colombatti, L. Chieco-Bianchi

## Abstract

To investigate whether AKR spontaneous leukaemogenesis is associated with a reduction in functional activity of T lymphocytes, the PHA response of AKR blood cells at different ages up to and including the preleukaemic period was studied. No significant differences were observed among young, adult and preleukaemic donors. In addition, the in vitro and in vivo AKR lymphocyte functions were compared with those of CBA lymphocytes by means of their response to stimulation with T and B lymphocyte selective mitogens (PHA, Con A and LSP respectively), and their response to immunization with thymus dependent (SRBC) or independent (LPS) antigens. We observed in vitro that while the B lymphocytes responded normally to mitogen, an intrinsic hyporeactivity to mitogens characterizes the T lymphocytes. Moreover, AKR mice exhibited a reduced in vivo response to both thymus dependent and independent antigens.


*Br. J. Cancer* (1975) **32**, 331

### *IN VITRO* AND *IN VIVO* EVALUATION OF T AND B LYMPHOCYTE FUNCTIONS IN AKR MICE

D. COLLAVO, G. BIASI, A. COLOMBATTI AND L. CHIECO-BIANCHI

*From the Laboratory of Experimental Oncology, Institute of Pathological Anatomy, University of Padova, Italy*

Received 15 May 1975. Accepted 29 May 1975

**Summary.—To investigate whether AKR spontaneous leukaemogenesis is associated with a reduction in functional activity of T lymphocytes, the PHA response of AKR blood cells at different ages up to and including the preleukaemic period was studied. No significant differences were observed among young, adult and preleukaemic donors. In addition, the *in vitro* and *in vivo* AKR lymphocyte functions were compared with those of CBA lymphocytes by means of their response to stimulation with T and B lymphocyte selective mitogens (PHA, Con A and LSP respectively), and their response to immunization with thymus dependent (SRBC) or independent (LPS) antigens.**

**We observed *in vitro* that while the B lymphocytes responded normally to mitogen, an intrinsic hyporeactivity to mitogens characterizes the T lymphocytes. Moreover, AKR mice exhibited a reduced *in vivo* response to both thymus dependent and independent antigens.**

IN 1960, Old and his co-workers reported that mice infected with Friend murine leukaemia virus (F-MuLV) displayed a diminished antibody producing capacity towards sheep red blood cells (SRBC). Since then, the immunodepressive effects exerted by MuLVs in mice and rats subsequently immunized with a variety of antigens have been well documented (for review articles, see Notkins, Mergenhagen and Howard, 1970; Dent, 1972; Friedman and Ceglowski, 1973). In most experimental models investigated, depression of immune reactivity was evident long before clinical onset of the leukaemic disease. Consequently, it was postulated that an important factor contributing to the pathogenesis of haematopoietic malignancies could be a decrease in host immune capacity operated by the oncogenic agents themselves. However, AKR mice, which are considered the natural carriers of endogenous Gross (G) MuLV, exhibit a severely depressed immune response only when frankly leukaemic (Metcalf and Moulds, 1967). In addition, during the AKR preleukaemic period, immune reactivity has been reported as either normal or decreased (Metcalf and Moulds, 1967; Hargis and Malkiel, 1972; Doré, Schneider and Mathé, 1969). Since the leukaemias which develop spontaneously in adult AKR mice originate in the thymus (Metcalf, 1966), it is likely that the thymus derived (T) lymphocytes represent the actual targets for virus induced neoplastic conversion. Moreover, in many instances the cell surface marker for T cells, the $\theta$ antigen, has been found on AKR leukaemic lymphocytes (Grey *et al.*, 1972). Thus, if a deficiency in AKR immune reactivity is produced early in life as a consequence of G-MuLV infection, the T cell functions should be primarily affected.

In previous preliminary experiments (Biasi *et al.*, 1974), we had observed that the response of AKR lymphocytes *in vitro* to phytohaemagglutinin (PHA), a T cell selective mitogen, did not vary with increasing donor age, up to and including the preleukaemic period, and that AKR

lymphocytes were considerably less stimulated by PHA than CBA lymphocytes. The present study reports further observations regarding the *in vitro* and *in vivo* functional activity of both T and bursa equivalent derived (B) lymphocytes of AKR mice.

### MATERIALS AND METHODS

*Mice.*—Inbred AKR, CBA and CBAT6T6 (hereafter called T6) mice from our colony were used throughout.

*Preparation of cell suspensions.*—Blood samples were collected by heart section according to Festenstein (1968). Citrated blood was defibrinated on glass beads by adding Ca gluconate and then mixed 1 : 1 with Plasmagel (Roger Bellon, Neuilly-Paris, France) in order to separate the erythrocytes. The lymphocyte-rich supernatant was centrifuged at 600 *g* for 10 min and cells were resuspended in RPMI 1640 Medium (Eurobio, Paris, France) containing 15% heat inactivated foetal calf serum (FCS) (Grand Island Biol. Co., Grand Island, N.Y., U.S.A.) and antibiotics. Spleen and lymph nodes were minced and pressed through a multilayer nylon sieve using complete medium as diluent and then washed twice at 4°C. The number of viable cells was determined by the Eosin Y exclusion method. No FCS was added to the medium when cells were used for haemolytic plaque forming cell assay.

*Blood lymphocyte cultures.*—(a) Karyotype analysis assay (Doenhoff *et al.*, 1970): Mixed cultures of AKR and T6 cells contained various proportions of each cell type; the final concentration in all cultures was $2 \times 10^6$ cells/ml medium. Sufficient reconstituted PHA (Wellcome, Beckenham, England) was then added to each culture to reach a final dilution of 1/160. Preliminary studies had shown this dose to be optimal for achieving maximum response. The same batch of PHA was used throughout (Lot No. K4418). Culture vials consisted of 2 ml disposable plastic tubes (Stayne Continental S.A., Jumet Diarbois, Belgium). Cultures were incubated at 37°C in a 5% $CO_2$ moist atmosphere for 72 h. Twelve h before harvesting, Colcemid (Ciba, Basel, Switzerland) was added to each culture to a final concentration of $10^{-7}$ mol/l. The cells were then transferred to a 1% hypotonic Na citrate solution and the preparation of metaphases for karyotype analysis was carried out according to Ford (1966). Two slides were examined from each culture and 50–100 metaphases per slide were analysed.

(b) Quantitation of DNA synthesis: AKR and T6 cells were cultured separately ($2 \times 10^6$ cells/ml medium) as described above. Twelve h before harvesting, 2 $\mu$Ci thymidine-methyl-$^3$H($^3$H-TdR, specific activity 2·0 Ci/mmol, NEN, Frankfurt, Germany) were added to each culture. Cultures were processed and $^3$H-TdR uptake was calculated by liquid scintillation counting (Doenhoff *et al.*, 1970).

*Spleen cell cultures.*—$2 \times 10^6$ cells were cultured in 1 ml medium as described above. Triplicate sets received PHA at a final concentration 1 : 100 or 2 $\mu$g concanavalin-A (Con A, Calbiochem, San Diego, Calif., U.S.A.) or 25 $\mu$g lipopolysaccharide B *E. coli* (LPS, 055:B5 Difco, Detroit, Mich., U.S.A.). All doses were calculated to give a maximum mitogenic response. Triplicate sets without mitogens served as controls. Cultures were then processed as described in (b).

*Antigens.*—SRBC (Sclavo, Siena, Italy) were washed twice with saline and $4 \times 10^8$ cells in 0·1 ml saline were injected i.p. 0·02 mg LPS in 0·1 ml saline was injected i.p.

*Detection of antibody producing cells.*—The number of direct haemolytic plaque forming cells (PFC) against SRBC was determined on Day 4 and 5 following immunization, according to Jerne, Nordin and Henry (1963). The number of direct PFC against LPS coated SRBC was determined on Day 3, 4 and 5. SRBC coating with LPS was achieved as described by Möller (1965). Agar plates containing the cell suspensions adjusted to give about 50–100 PFC/plate were incubated at 37°C for 1 h, after which 3 ml of 1 : 10 guinea-pig serum (Sclavo, Siena, Italy) were added. The plates were incubated again at 37°C for 1 h in order to developed haemolytic plaques. PFC were counted at $\times 10$ magnification.

*Statistical analysis.*—Student's *t* test was used to compare the experimental results.

### RESULTS

### *Response of AKR blood lymphocyte cultures to PHA stimulation*

Karyotype analysis was chosen because it requires a low number of cells and can

TABLE I.—*Relationship between Number of AKR Cells in Culture and Resulting Mitosis Following PHA Stimulation**

| No. of cells in culture × 10⁻⁶ AKR | T6 | Expected AKR/T6 Mitosis ratio† | Obtained AKR/T6 Mitosis ratio | Corrected AKR/T6 Mitosis ratio‡ |
|---|---|---|---|---|
| 1·4 | 0·6 | 2·33 | 1·60 | 0·69 |
| 1·0 | 1·0 | 1·00 | 0·72 | 0·72 |
| 0·8 | 1·2 | 0·66 | 0·49 | 0·73 |
| 0·6 | 1·4 | 0·43 | 0·30 | 0·70 |

* Each value represents the mean of a single experiment performed with 3 different pools of AKR and T6 blood cells.

† Expected mitosis ratio refers to the value that would have been obtained if the 2 cell populations had responded identically to PHA stimulation.

‡ Values were corrected by dividing the obtained AKR/T6 mitosis ratio by the ratio of AKR/T6 cells in culture.

be carried out on single animals. Moreover, with this assay variations in the number of PHA responsive cells can be quantitated very precisely. As reported in Table I cultures were set up in which the AKR/T6 cell concentration varied. The results indicate that AKR cells are less responsive to PHA than T6 and consequently the AKR/T6 mitosis ratio obtained fell below the mitosis ratio expected on the basis of a straight cell count. However, by correcting each AKR/T6 mitosis ratio with its respective AKR/T6 cell ratio, a value was obtained which was fairly similar for all the cell concentrations used. Therefore, if the cell populations tested present a variation in the PHA responsive cell number, a proportional variation in the number of mitoses is detectable.

It was necessary to exclude the possibility that a mixed lymphocyte reaction (MLR) between AKR and T6 cells might have modified the results (Festenstein, 1973) even though the incubation time was relatively brief. Thus, we correlated $^{3}$H-TdR uptake of AKR and T6 cells cultured separately with the number of mitoses scored when cells from the same suspensions were cultured together. Table II reports the results obtained employing a single T6 blood pool and 4 different AKR blood pools. In all the blood samples tested, there is a good agreement between the AKR/T6, $^{3}$H-TdR uptake ratio and the mitosis ratio. Accordingly, if an MLR took place, it did not interfere with the PHA response of the 2 cell populations.

Experiments were then performed to determine the percentage of AKR blood lymphocytes from 1, 2–4 and 6 month old mice which responded by mitosis to PHA stimulation *in vitro*. The results are based on the average of 8 experiments, carried out by mixing cells from individual AKR donors with the blood pool obtained from a single group of 3-month old T6 donors. As appears in Fig. 1, the percentage of AKR mitoses remained fairly stable with ageing. In addition, little difference in PHA reactivity among individual AKR mice was noted in all 3 age groups. Finally, as in the above experi-

TABLE II.—*Correlation between PHA Responses of AKR Blood Cells Evaluated by DNA Synthesis and by Karyotype Analysis Methods*

| | | Sample tested* 1 | 2 | 3 | 4 |
|---|---|---|---|---|---|
| $\Delta$† ct/min × $10^{-3}$ | AKR | 13·4 | 15·4 | 13·3 | 11·0 |
| | T6 | 19·6 | 19·6 | 19·6 | 19·6 |
| AKR/T6 ratio | | 0·68 | 0·78 | 0·67 | 0·56 |
| Mitosis‡ | AKR | 40 | 42 | 39 | 37 |
| | T6 | 60 | 58 | 61 | 63 |
| AKR/T6 ratio | | 0·67 | 0·72 | 0·64 | 0·58 |

* 4 pools of blood cells from groups of 3 AKR mice each were compared with a single blood pool from 10 T6 donors.

† $\Delta$ = difference between the average of $^{3}$H-TdR uptake in PHA stimulated and unstimulated triplicate cultures.

‡ AKR and T6 cells were cultured together in equal proportions.

FIG. 1.—Percentage of AKR cells responding by mitosis to PHA stimulation in blood cultures from different age group donors. AKR and T6 cells were cultured in equal proportions. The number of AKR donors is given in parentheses. Each value represents the mean ± standard deviation (s.d.).

ments, AKR blood lymphocytes exhibited a lower PHA response and the mean of AKR/T6 mitoses was 0·82 when the 45 mice tested were considered all together.

No studies were made on lymphocytes of leukaemic AKR mice as they are known to have high spontaneous replication rate.

*Response of AKR and CBA spleen cell cultures to Con A, PHA and LPS stimulation*

The response of AKR spleen lymphocytes to different mitogens was compared with that of CBA mice. As reported in Table III, spleen cells from individual mice were stimulated with Con A, PHA (T cell mitogens) and LPS (B cell mitogen). Following Con A and PHA stimulation, $^{3}$H-TdR uptake and stimulation index (SI) in AKR mice was significantly lower than in CBA mice ($P < 0{\cdot}01$). On the other hand, no differences were found between these 2 strains following LPS stimulation, indicating that although AKR T lymphocytes respond poorly, B lymphocytes are fully responsive. Moreover, a similar number of $\theta$ positive cells was found in blood and peripheral lymphoid organs of both AKR and CBA mice (Collavo *et al.*, 1975). Accordingly, T mitogen hyporeactivity detected in AKR mice cannot be ascribed to a lower T cell number.

In vivo *response to SRBC and LPS immunization*

It was of interest to determine if our *in vitro* observations reflected the *in vivo*

TABLE III.—*Response of AKR and CBA Spleen Cultures to Con A, PHA and LPS Stimulation**

| Strain | Unstimulated | Con A stimulated | SI | PHA stimulated | SI | LPS stimulated | SI |
|---|---|---|---|---|---|---|---|
| AKR | 1921 ± 189 | 11916 ± 941 | 6·2 | 5095 ± 776 | 2·7 | 9658 ± 1222 | 5·1 |
| CBA | 1741 ± 310 | 20978 ± 1603 | 12·0 | 8587 ± 1669 | 5·3 | 9791 ± 1758 | 5·8 |

* DNA synthesis determined by $^3$H-TdR uptake in ct/min.

Each value represents the mean ± standard error s.e. of triplicate cultures from 5 different donors.

SI = Stimulation index: ratio of ct/min in stimulated/unstimulated cultures.

FIG. 2.—Direct PFC production following SRBC immunization in AKR and CBA mice. Spleen cells from 7 AKR (shaded columns) and 7 CBA (clear columns) were assayed individually. Each value represents the mean ± s.e.

pattern of response. AKR and CBA mice were injected i.p. with thymus dependent (SRBC) and independent (LPS) antigens. At different intervals following the immunization the spleen direct PFC against SRBC and LPS coated SRBC were evaluated. As shown in Fig. 2, SRBC immunized AKR mice on Day 4 produced less PFC (evaluated both per $10^6$ cells and per spleen) than CBA, and even less on Day 5 ($P < 0{\cdot}01$ and $P < 0{\cdot}001$ respectively).

A greater difference between the 2 strains was observed following LPS injection (Fig. 3). AKR mice produced a much lower number of PFC on different days (4, 5, 6), evaluated both per $10^6$ cells and per spleen ($P < 0{\cdot}001$). Thus, *in vivo* data suggest that the immunological deficit in AKR mice involves the response to both thymus dependent and independent antigens.

### DISCUSSION

Our previous (Biasi *et al.*, 1974) and present results indicate that the response to PHA stimulation of AKR blood and spleen cells from young, adult and pre-leukaemic mice is constant. While our data are in line with studies reporting

FIG. 3.—Direct PFC production following LPS immunization in AKR and CBA mice. Spleen cells from 7 AKR (shaded columns) and 7 CBA (clear columns) were assayed individually. Each value represents the mean ± s.e.

that antibody production to SRBC (Metcalf and Moulds, 1967) and GVH reactivity (Hargis and Malkiel, 1972) are not reduced in preleukaemic AKR mice, there is no general agreement on this topic. Skin graft rejection, GVH reaction (Doré *et al.*, 1969) and response to PHA and Con A (Nagaya, 1973; Zatz, Goldstein and White, 1973) have also been reported impaired in preleukaemic AKR mice.

Genetic dissimilarities, leading to different expression of MuLV group specific (gs) antigen and spontaneous leukaemia incidence among the various AKR sublines (Acton *et al.*, 1973), might explain the discrepancies between our results and those of others. AKR mice employed in this study derive from the colony of the Laboratory of Genetics, Radium Institute, Paris (Dr Rudali) and are characterized by a high MuLV gs expression and an 80–90% spontaneous leukaemia incidence.

Comparing adult AKR mice with mice of other strains, several workers have reported a defect in T cell function (Hays, 1972; Frey-Wettstein and Hays, 1970; Zatz *et al.*, 1973). In the present study, AKR immune reactivity was compared with that of CBA mice. These mice were chosen as controls because they exhibit a low spontaneous leukaemia incidence, share the same H-$2^k$ haplotype and possess a similar amount of peripheral $\theta$-positive cells. Following *in vitro* exposure to mitogens, the response of AKR spleen lymphocytes to Con A and PHA was markedly reduced, whereas it was unchanged after LPS stimulation. Therefore, it seems likely that in AKR mice an intrinsic hyporeactivity to selective mitogens exists and it seems to affect the T cell population only.

The *in vivo* evaluation of immune reactivity is in partial agreement with our *in vitro* findings. In comparison with CBA, the lower AKR response to SRBC could be interpreted in terms of a functional deficiency in a T cell co-operative activity. It is difficult to explain the reduced response to immunization with

LPS, since the *in vitro* response was normal. However, although immune and mitogenic responsiveness to LPS seem to share a common genetic regulatory mechanism (Watson and Riblet, 1974), evidence exists that the antigenic and mitogenic properties of LPS are quite distinct (Andersson, Sjöberg and Möller, 1973; Chiller *et al.*, 1973).

The question still remains if AKR immune deficiency is caused by an endogenous G-MuLV infection and the consequent neoplastic transformation of the lymphoid cells. The observation that the immune defect in T cells is present in mice of different age groups, thus showing no correlation with the virus titre, and that it affects also the B cells which are rarely involved in neoplastic conversion, does not favour this hypothesis. Furthermore, we have recently observed that CBA mice neonatally infected with passage A G-MuLV do not show any reduction in PHA and LPS lymphocyte response *in vitro*, nor a reduced *in vivo* antibody production to SRBC or LPS antigens when compared with normal control (Collavo *et al.*, 1975). Therefore, it seems reasonable to conclude that the full expression of endogenous G-MuLV and the resulting high leukaemia incidence are merely the consequence of a complex intrinsic defect which alters the immune reactions in AKR mice.

This work was supported in part by grants from the Consiglio Nazionale delle Ricerche, Roma, and the Associazione per la Promozione delle Ricerche sul Cancro, Milano.

We thank Drs A. J. S. Davies and M. Doenhoff for their helpful advice and criticism. The skilful technical assistance of Silvio Mezzalira is gratefully acknowledged.

### REFERENCES

ACTON, R. I., BLANKENHORN, E. P., DOUGLAS, T. C., OWEN, R. D., HILGERS, J., HOFFMAN, H. A. & BOYSE, E. A. (1973) Variations among Sublines of Inbred AKR mice. *Nature, New Biol.*, **245**, 8.

ANDERSSON, J., SJÖBERG, O. & MÖLLER, G. (1972) Mitogens as Probes for Immunocyte Activation and Cellular Cooperation. *Transplantn Rev.*, **11**, 131.

BIASI, G., COLLAVO, D., BIASI, A. & CHIECO-BIANCHI, L. (1974) Evaluation of T Lymphocyte Reactivity in Leukemia Prone AKR Mice. In *Characterization of Human Tumors*, Ed. W. Davis and C. Maltoni. Amsterdam: Excerpta Medica. p. 261.

CHILLER, J. M., SKIDMORE, B. J., MORRISON, D. C. & WEIGLE, W. O. (1973) Relationship of the Structure of Bacterial Lipopolysaccharide to its Function in Mitogenesis and Adjuvanticity. *Proc. natn. Acad. Sci., U.S.A.*, **70**, 219.

COLLAVO, D., BIASI, G., COLOMBATTI, A. & CHIECO-BIANCHI, L. (1975) Effect of Endogenous and Exogenous Murine Leukemia Virus Infection on Immunologic Response. *Eur. J. Cancer*, **11**, 443.

DENT, P. B. (1972) Immunodepression by Oncogenic Viruses. *Prog. med. Virol.*, **14**, 1.

DOENHOFF, M. J., DAVIES, A. J. S., LEUCHARS, E. & WALLIS, V. (1970) The Thymus and Circulating Lymphocytes of Mice. *Proc. R. Soc. Lond. B.*, **176**, 69.

DORÉ, J. F., SCHNEIDER, M. & MATHÉ, G. (1969) Réactions immunitaires chez les souris AKR leucémiques ou préleucémiques. *Rev. franç. Étud. clin. Biol.*, **14**, 1003.

FESTENSTEIN, H. (1968) Mouse Peripheral Blood Lymphocytes in Culture. *Lancet*, i, 182.

FESTENSTEIN, H. (1973) Immunogenetics and Biological Aspects of *in vitro* Lymphocyte Allotransformation (MLR) in the Mouse. *Transplantn Rev.*, **15**, 62.

FORD, C. E. (1966) The Use of Chromosome Markers. In *Tissue Grafting and Radiation.* Ed. H. S. Micklem and J. F. Loutit. New York: Academic Press. Appendix 1, p. 197.

FREY-WETTSTEIN, M. & HAYS, E. F. (1970) Immune Response in Preleukemic Mice. *Infect. Immun.*, **2**, 398.

FRIEDMAN, H. & CEGLOWSKI, W. S. (1973) Cellular Immunity and Leukemia Virus Infection. In *Virus Tumorigenesis and Immunogenesis.* Ed. W. S. Ceglowski and H. Friedman. London: Academic Press. p. 299.

GREY, H. M., COLÓN, S., CAMPBELL, P. & RABELLINO, E. (1972) Immunoglobulins on the Surface of Lymphocytes. V. Quantitative Studies on the Question whether Immunoglobulins are Associated with T Cells in the Mouse. *J. Immun.*, **109**, 776.

HARGIS, B. J. & MALKIEL, S. (1972) The Immunocapacity of the AKR Mouse. *Cancer Res.*, **32**, 291.

HAYS, E. F. (1972) Graft versus Host Reactions and the Viral Induction of Mouse Lymphoma. *Cancer Res.*, **32**, 270.

JERNE, N. H., NORDIN, A. A. & HENRY, C. (1963) The Agar Plaque Technique for Recognizing Antibody-producing Cells. In *Cell Bound Antibodies.* Ed. H. Koprowski and B. Amos. Philadelphia: Wistar Institute Press. p. 107.

METCALF, D. (1966) Histologic and Transplantation Studies on Preleukemic Thymus of the AKR Mouse. *J. natn. Cancer Inst.*, **37**, 425.

METCALF, D. & MOULDS, R. (1967) Immune Responses in Preleukemic and Leukemic AKR Mice. *Int. J. Cancer*, **2**, 53.

MÖLLER, G. (1965) 19s Antibody Production against

Soluble LPS Antigens by Individual Lymphoid Cells *in vitro*. *Nature, Lond.*, **207**, 1166.

NAGAYA, H. (1973) Thymus Function in Spontaneous Lymphoid Leukemia. II. *In vitro* Response of "Preleukemic" and Leukemic Thymus Cells to Mitogens. *J. Immun.*, **111**, 1052.

NOTKINS, A. L., MERGENHAGEN, S. E. & HOWARD, R. J. (1970) Effect of Virus Infections on the Function of the Immune System. *A. Rev. Microbiol.*, **24**, 525.

OLD, L. J., CLARKE, D. A., BENACERRAF, B. & GOLDSMITH, M. (1960) The Reticuloendothelial System and the Neoplastic Process. *Ann. N.Y. Acad. Sci.*, **88**, 264.

WATSON, J. & RIBLET, R. (1974) Genetic Response to Bacterial Lipopolysaccharydes in Mice. I. Evidence for a Single Gene that Influences Mitogenic and Immunogenic Responses to Lipopolysaccharydes. *J. exp. Med.*, **140**, 1147.

ZATZ, M. M., GOLDSTEIN, A. L. & WHITE, A. (1973) Lymphocyte Populations of AKR/J Mice. I. Effect of Ageing on Migration Pattern, Response to PHA and Expression of Theta Antigen. *J. Immun.*, **111**, 1514.